# The Cell Cycle Timing of Centromeric Chromatin Assembly in Drosophila Meiosis Is Distinct from Mitosis Yet Requires CAL1 and CENP-C

**DOI:** 10.1371/journal.pbio.1001460

**Published:** 2012-12-27

**Authors:** Elaine M. Dunleavy, Nicole L. Beier, Walter Gorgescu, Jonathan Tang, Sylvain V. Costes, Gary H. Karpen

**Affiliations:** 1 Department of Genome Dynamics, Life Sciences Division, Lawrence Berkeley National Laboratory, and Department of Molecular and Cell Biology, University of California Berkeley, Berkeley, California, United States of America; 2 Department of Cancer and DNA Damage Responses, Life Sciences Division, Lawrence Berkeley National Laboratory, Berkeley, California, United States of America; University of Cambridge, United Kingdom

## Abstract

CENP-A (CID in flies) is the histone H3 variant essential for centromere specification, kinetochore formation, and chromosome segregation during cell division. Recent studies have elucidated major cell cycle mechanisms and factors critical for CENP-A incorporation in mitosis, predominantly in cultured cells. However, we do not understand the roles, regulation, and cell cycle timing of CENP-A assembly in somatic tissues in multicellular organisms and in meiosis, the specialized cell division cycle that gives rise to haploid gametes. Here we investigate the timing and requirements for CID assembly in mitotic tissues and male and female meiosis in *Drosophila melanogaster*, using fixed and live imaging combined with genetic approaches. We find that CID assembly initiates at late telophase and continues during G1 phase in somatic tissues in the organism, later than the metaphase assembly observed in cultured cells. Furthermore, CID assembly occurs at two distinct cell cycle phases during male meiosis: prophase of meiosis I and after exit from meiosis II, in spermatids. CID assembly in prophase I is also conserved in female meiosis. Interestingly, we observe a novel decrease in CID levels after the end of meiosis I and before meiosis II, which correlates temporally with changes in kinetochore organization and orientation. We also demonstrate that CID is retained on mature sperm despite the gross chromatin remodeling that occurs during protamine exchange. Finally, we show that the centromere proteins CAL1 and CENP-C are both required for CID assembly in meiosis and normal progression through spermatogenesis. We conclude that the cell cycle timing of CID assembly in meiosis is different from mitosis and that the efficient propagation of CID through meiotic divisions and on sperm is likely to be important for centromere specification in the developing zygote.

## Abstract 2

The centromeric histone CENP-A is incorporated at different cell cycle phases during somatic mitosis, meiosis I and meiosis II in *Drosophila melanogaster*.

## Author Summary

Centromeres are regions of eukaryotic chromosomes that recruit the kinetochores and are essential for faithful segregation of DNA during all cell divisions. The centromere-specific histone H3 variant CENP-A accumulates at the centromere, defining this region, and is maintained throughout cellular generations by epigenetic mechanisms in most eukaryotes. Previous studies have discovered many factors regulating both the maintenance and assembly of CENP-A at centromeres during mitosis in cultured cells, but the mode of regulation of CENP-A assembly during meiosis and mitosis in animal tissues is unknown. In this study, we use *Drosophila melanogaster* as an organismal model to investigate the timing and requirements for assembly of CID, the fly CENP-A homolog. We find that that CID is loaded at centromeres during telophase/G1 phase in brain stem and nonstem cells. In male meiosis, CID is loaded in two phases, during the first stages of meiosis I and after the second meiotic division. Meiosis I loading time is also conserved in females. We also report an unprecedented drop in CID levels after meiosis I and before meiosis II, which correlates with the timing of kinetochore reorientation. Additionally, we find that two essential centromere proteins (CAL1 and CENP-C) are necessary for CID assembly and chromosome segregation during meiosis. Our data demonstrate novel differential timing for CENP-A assembly during mitosis and meiosis in the whole organism.

## Introduction

Centromeres are key regions of eukaryotic chromosomes that ensure proper chromosome segregation during cell divisions. In most eukaryotes, centromere identity is defined epigenetically by the presence of a centromere-specific histone H3 variant CENP-A (CID in flies, CENH3 in some organisms) [1]. Improper regulation of CENP-A assembly leads to aberrant segregation of chromosomes, aneuploidy, and cell death [2]–[5]. Relevance to human disease comes from observations that CENP-A is overexpressed and can misincorporate throughout chromatin in human cancers [6],[7], that most human cancers display severe aneuploidy [8], and that CID overexpression results in formation of ectopic centromeres and aneuploidy [3],[4].

Centromere propagation requires assembly of new chromatin components after they are diluted 2-fold by DNA replication and segregation of preexisting nucleosomes to sister centromeres. In recent years, great insight into how centromeres are reproducibly propagated during the mitotic cell cycle has emerged from studies investigating the cell cycle timing of CENP-A assembly [9]. A common theme has emerged for multicellular eukaryotes; unlike canonical histones, which are assembled concurrently with DNA replication, CENP-A nucleosome deposition occurs after centromeric DNA replication, during mitosis or G1 phase. In human tissue culture cells and Xenopus egg extracts, CENP-A assembly occurs during late telophase/early G1 phase [10]–[12]. In Drosophila, CID is assembled at metaphase in tissue culture cells [13] and anaphase in embryonic syncytial divisions [14]. Interestingly, anaphase loading was not observed in late embryonic stages in flies, and the exact timing of CID assembly during these or later developmental stages is unknown [14]. Thus, the timing of CENP-A assembly, and likely its regulation, differs between organisms, as well as developmental stages in the same organism. Indeed, aside from investigations in single cell eukaryotes, cells in culture, and unusual syncytial divisions (featuring rapid S and M phases with no gap phases), the cell cycle timing of CENP-A assembly in somatic mitotic tissues in animals has not yet been determined.

Additional biochemical and genetic approaches in single cell eukaryotes or cultured cells have identified many proteins critical for CENP-A assembly in mitosis. In humans, CENP-A deposition is mediated by its chaperone and assembly factor HJURP [15]–[18], while the HJURP homolog Scm3 performs these functions in yeasts [19]–[23]. In Drosophila tissue culture cells and embryos, the putative HJURP functional homolog CAL1 and the constitutive centromere component CENP-C are both required for CID localization at centromeres, and CAL1, CENP-C, and CID co-immunoprecipitate in vivo [13],[24]–[26]. Moreover, CAL1 has distinct binding domains for both CID and CENP-C, and its low levels prevent excess CID incorporation at mitotic centromeres [25]. There is also accumulating evidence that CENP-A assembly is tightly coupled to mitotic cell cycle activities, including activation of the Anaphase Promoting Complex/Cyclosome (APC/C), degradation of the mitotic regulator Cyclin A (CycA) in flies [13],[24], and inhibition of cyclin-dependent kinase (CDK) activities in mammalian cell lines [27]. However, the precise mechanisms and targets of cell cycle control of centromere assembly remain to be elucidated.

In contrast to mitosis, the functional requirements, regulation, and timing of CENP-A assembly in the specialized meiotic divisions that occur during gametogenesis are largely unknown. Meiosis produces haploid gametes (eggs and sperm) and encompasses two distinct types of chromosome segregation. In meiosis I, sister chromatids attach to a common kinetochore and mono-orient, segregating homologous chromosomes, while in meiosis II, sister chromatids bi-orient and segregate equationally, similar to mitosis. In *C. elegans*, normal levels of CENP-A are not required for meiosis, and CENP-A is removed from chromosomes during female meiosis II [28] and is also absent from mature sperm [29]. CENP-A is required for proper meiotic segregation in *Arabidopsis*, but meiosis-specific factors appear to facilitate CENP-A assembly [30],[31]. Thus, CENP-A assembly and propagation appear to be differentially regulated in mitosis and meiosis, both within an organism and between different species. Furthermore, in most eukaryotes, CENP-A is one of the few histones retained on mature sperm [32]–[35]. Presumably, marking the site of CENP-A assembly on paternal chromosomes is crucial for centromere inheritance and propagation in early embryonic divisions.

Here we investigate the cell cycle timing and regulation of CID assembly in animal tissues, specifically *Drosophila melanogaster* larval brains and male and female meiosis. We find that new CID is assembled at centromeres in late telophase and continues into early G1 phase in somatic mitoses, later than observed in early embryos (anaphase) and cultured cells (metaphase) [13],[14]. In meiosis, CID is assembled at two cell cycle phases: prophase of meiosis I and after exit from meiosis II, in spermatids. We also observe an unprecedented decrease in CID levels between the end of meiosis I and the beginning of meiosis II. Additionally, we show that CID assembly in meiosis requires CAL1 and CENP-C. We conclude that the cell cycle timing and dynamics of CID assembly in meiosis are different from mitosis and also differ between mitotic cells in culture and in the animal.

## Results

### CID Is Assembled at Centromeres During Telophase/G1 Phase in Larval Brain Mitoses

**Figure 1:**
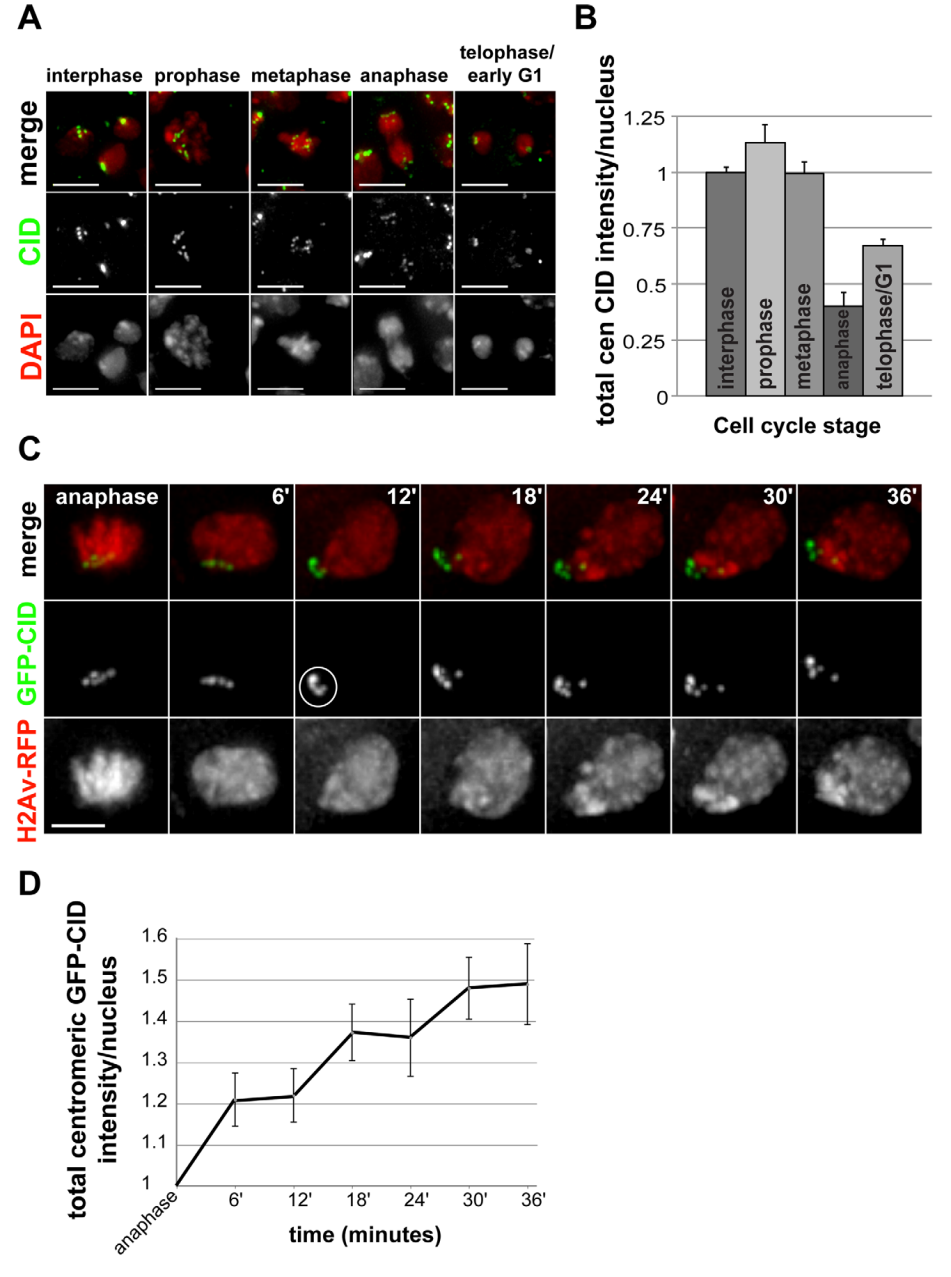
Cell cycle timing of CID assembly in mitotic tissues. (A) Changes in the amount of CID at centromeres during mitosis in nonstem brain cells. Larval brains were fixed and stained with anti-CID antibody (green) and DNA is stained with DAPI (red). Scale bar: 5 µM. (B) Quantification of total centromeric CID fluorescence intensity per nucleus in stages of mitosis in dividing nonstem brain cells. Condensed chromatin at metaphase and anaphase results in a reduction in antibody penetration. Bars, standard errors. Values are normalized to the interphase average. *N* = 318 total cells. *N* = 171, interphase; 35, prophase; 52, metaphase; 21, anaphase; 39, telophase/early G1. Scale bar: 5 µM. (C) Live imaging of a nonstem brain cell from anaphase into early G1 phase expressing GFP-CID (green) and the chromatin marker H2Av-RFP (red). Time elapsed is shown in minutes. Circle indicates the initiation of CID assembly between 6 and 12 min after anaphase onset. Scale bar: 3 µM. (D) Quantification of total centromeric GFP-CID fluorescence intensity per nucleus from live imaging of dividing nonstem brain cells in larvae (*n* = 9 movies). Time elapsed in minutes after anaphase onset is shown on the *x*-axis, and fold increase in total centromeric GFP-CID intensity per nucleus is shown on the *y*-axis. Bars, standard errors.

Current insights into the cell cycle timing of CENP-A assembly have come from experiments in tissue culture cells, single cell eukaryotes, or the unusual syncytial divisions in embryos (S and M phases with no gap phases). To elucidate the timing of CENP-A assembly in mitotic cells in animal somatic tissues, we stained dividing cells in larval brains with anti-CID antibody and measured total CID intensity at centromeres using custom software (see Materials and Methods). In brain nonstem cells, we found that levels of CID per cell are relatively constant throughout interphase, prophase, and metaphase; are reduced by half at anaphase; increase in intensity beginning at late telophase/early G1 phase (Figure 1A and 1B); and have doubled by early S phase (Figure S1A). Total CID intensity measured at early G1 phase was less than observed in interphase, implying that loading continues through G1, as previously reported in human cell lines [36],[37].

To exclude the possibility that changes in CID intensity were due to differences in antibody staining or penetration at different cell cycle phases, we analyzed CID assembly using live imaging of larval brains expressing GFP-CID and the chromatin marker H2Av-RFP (Figure 1C and Movie S1) [14]. Using custom software (see Materials and Methods), we determined that total centromeric GFP-CID fluorescence intensity increases in daughter nuclei at telophase (approximately 6 to 12 min after anaphase onset) and continues during early G1 phase (Figure 1D). Notably, GFP-CID intensity increases by approximately 20% at late telophase and by 50% at 36 min past anaphase. Together, the fixed and live analyses of brain nonstem cells demonstrate that CID assembly initiates in telophase and continues in G1 phase, until centromeric CID levels double, replenishing the 2-fold CID dilution that occurs during DNA replication in S phase.

We also analyzed CID assembly in larval brain neuroblasts, large stem cells that undergo asymmetric divisions within a morphologically distinct circular niche (Figure S1B and Movie S2). Similar to brain nonstem cells, we observed that CID assembly occurs at telophase/early G1 phase in both the self-renewing mother stem cell and the daughter cell that later differentiates into a neuron. Interestingly, in five out of five movies analyzed, the initiation of CID assembly in the stem cell (3 min after anaphase onset, approximately 6 min earlier than in brain nonstem cells) precedes CID assembly in the daughter cell (9 min after anaphase onset, approximately the same time as in brain nonstem cells) (Figure S1B and Movie S2). Differential CID loading in neuroblasts was confirmed in fixed larval brains immunostained for CID (Figure S1C), where the mother and daughter cells in telophase displayed different CID levels.

We conclude that CID assembly in larval brain nonstem and stem cells begins during telophase and continues in G1 phase. This cell cycle assembly timing is similar to that reported for human tissue culture cells [10] and in Xenopus egg extracts [11],[12] but occurs later than observed in fly tissue culture cells (metaphase) and in embryos (anaphase) [13],[14].

### CID Is Assembled at Centromeres During Prophase of Meiosis I

**Figure 2:**
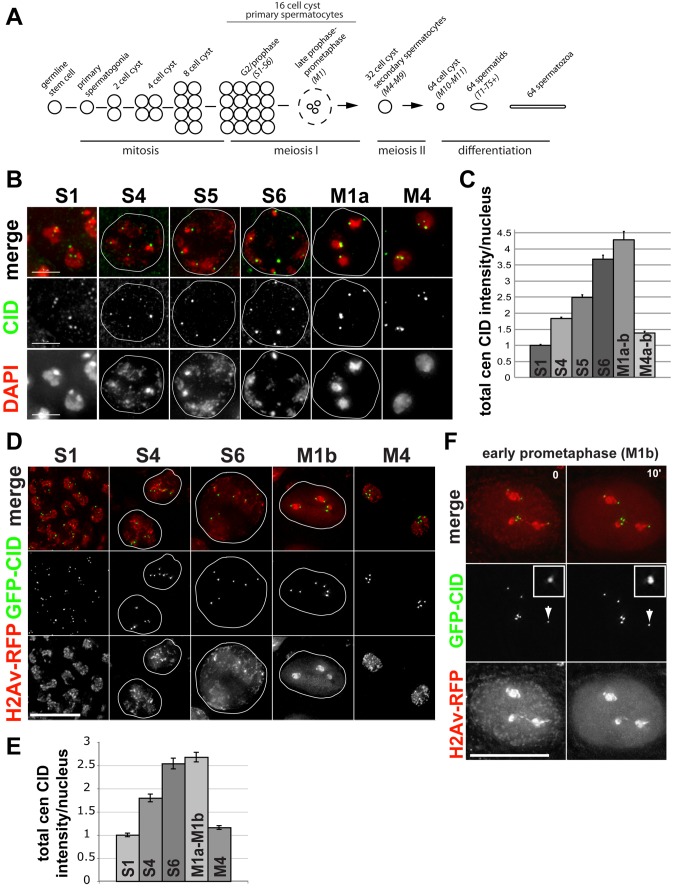
CID assembly in male meiosis I. (A) Spermatogenesis in *Drosophila* males. At the tip of the testes at the germinal proliferation center, a single germline stem cell divides mitotically producing a primary spermatogonial cell [62]. Primary spermatogonia complete four mitotic divisions and generate a cyst of 16 primary spermatocytes, which then replicate their DNA and undergo a 25-fold increase in volume in prophase I of meiosis, which is a developmentally specialized extended G2 phase (stages S1 to S6). At late prophase I of meiosis (M1), DNA condenses into three distinct domains, each corresponding to an autosomal pair. In the first meiotic division, each cell in a 16 cell cyst divides synchronously to form a cyst of 32 secondary spermatocytes (M4–M9). In the second meiotic division, secondary spermatocytes divide again to form a cyst of 64 spermatids (M10–M11). Further maturation and differentiation of spermatids (T1–T5+) over a period of days give rise to mature spermatozoa [38]. Standard nomenclature used is described in Cenci et al. [38]. (B) Changes in the amount of CID at centromeres during meiosis I. Larval testes were fixed and stained with anti-CID antibody (green), and DNA is stained with DAPI (red). CID localization in primary spermatocytes at stages S1, S4, S5, S6, and M1a of meiosis I and stage M4 of interphase II are shown. Outlines of nuclei are circled in white. Scale bar: 5 µM. (C) Quantification of total centromeric CID fluorescence intensity per nucleus in primary spermatocytes during stages S1 to S6 (prophase I), M1a–M1b (late prophase/early prometaphase I), and stage M4 (interphase II). Bars, standard errors. *N* = 157 total cells. *N* = 48, S1; 42, S4; 18, S5; 17, S6; 17, M1a–b; 32, M4a–b. Note that Figure 2C and 4B are from the same experiment and to the same scale, normalized to the initial S1 average intensity value. (D) Live imaging of primary spermatocytes expressing GFP-CID (green) and H2Av-RFP (red) at stages S1, S4, S6, and M1b (late prophase/early prometaphase) and M4 (interphase II) of meiosis I. Outlines of nuclei are circled in white. Scale bar: 15 µM. (E) Quantification of total centromeric GFP-CID fluorescence intensity per nucleus during S1 (*n* = 20), S4 (*n* = 18), and S6 (*n* = 26) of prophase I, M1a–M1b (late prophase/early prometaphase, *n* = 18), and M4a–M4b (interphase II, *n* = 23). Bars, standard errors. (F) Live imaging of a primary spermatocyte expressing GFP-CID (green) and H2Av-RFP (red) at early prometaphase of meiosis I (M1b). Time elapsed is in minutes. White arrows indicate centromeres shown in enlarged windows. Scale bar: 15 µM.

The cell cycle timing of CID assembly in meiosis is currently unknown and may differ from mitosis. The stages of male spermatogenesis encompass meiosis I, II, and subsequent differentiation steps that give rise to mature sperm (Figure 2A) [38]. We stained wild-type fixed late larval/prepupal testes with anti-CID antibody and quantified total centromeric CID fluorescence intensity per nucleus during these meiotic cell cycle stages using custom software (see Materials and Methods). We first focused our analysis on primary spermatocytes in 16 cell cysts that enter prophase of meiosis I; this is a developmentally specialized G2 phase that lasts for up to 90 hours, and is accompanied by a substantial increase in nuclear volume, followed by chromatin condensation at prometaphase I [38]. We observed a gradual increase in CID intensity from S1, S4, S5, and S6 stages up until late prophase/early prometaphase of meiosis I (M1a–b) (Figure 2B and 2C), indicating that CID assembly occurs over an extended period during prophase I. Surprisingly, we noted an approximate 4-fold increase in CID intensity during prophase I, larger than the predicted 2-fold increase expected to offset CID dilution during premeiotic S phase. We confirmed the gradual assembly of CID in prophase I by live imaging and quantification of GFP-CID intensity in primary spermatocytes expressing H2Av-RFP (Figure 2D and 2E). Consistent with results in fixed cells, we observed a gradual, greater than 2-fold increase in GFP-CID intensity at centromeres between stage S1 and early prometaphase of meiosis I (M1b) (Figure 2E). From time lapse imaging of cells in early prometaphase I, we observed one of the final CID assembly events (∼10% increase in GFP-CID intensity) in meiosis I, occurring in a relatively short, 10-min time window, approximately 40 min before condensed bivalents congress to the metaphase plate at prometaphase (Figure 2F and Movie S3).

Importantly, we did not detect any CID assembly after completion of meiosis I in fixed cells (compare stages M1a–M1b and M4a–M4b, Figure 2C) and further confirmed this result with live imaging (Figure 2D, Figure S2, and Movie S4). Surprisingly, in fixed and live cells we observe that CID intensity at stages M4a–M4b dropped by more than half of the amount present at stages M1a–M1b, indicating loss of centromeric CID after completion of meiosis I (Figure 2C and 2E). This decrease in CID levels in the absence of DNA replication is novel; CENP-A levels at centromeres have only been observed to decrease in response to replication and nucleosome segregation in S phase [10],[39]. At stages M4a–M4b, we were unable to detect distinct cell populations with high CID levels in either fixed or live cells, suggesting that the additional loss of CID after the first meiotic division occurs quickly after telophase.

**Figure 3:**
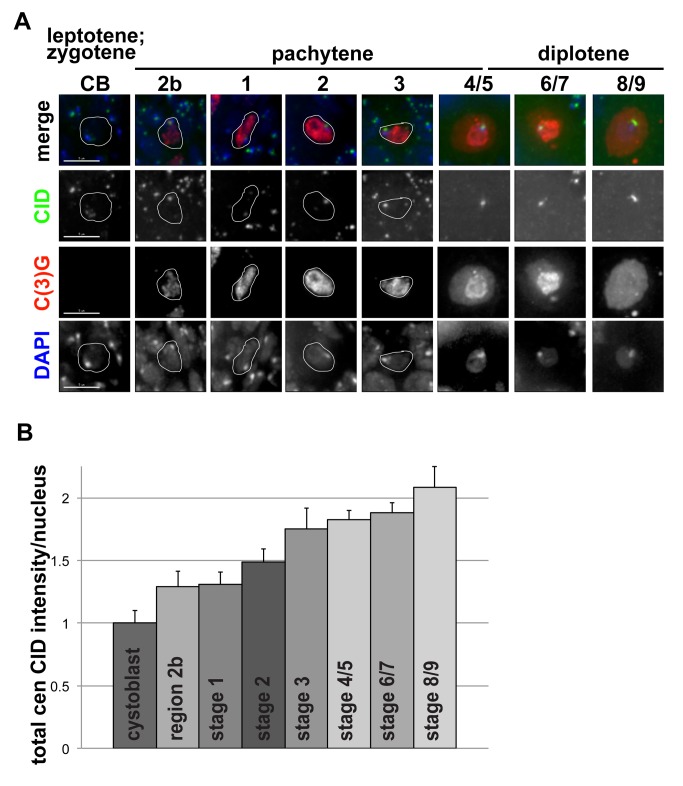
CID assembly in female meiosis I. (A) Changes in the amount of CID at centromeres during meiosis I in Drosophila females. Ovaries were fixed and stained with anti-CID antibody (green), anti-C(3)G antibody to mark the synaptonemal complex (red), and DNA is stained with DAPI (blue). Oocyte nuclei are circled in white. Staging is as described in [41],[63]. Scale bar: 5 µM. (B) Quantification of total centromeric CID fluorescence intensity per oocyte nucleus during prophase of meiosis I in Drosophila females. Bars, standard errors. *N* = 273 total cells. *N* = 15, cystoblast; 26, region 2b; 40, stage 1; 36, stage 2; 53, stage 3; 54, stage 4/5; 33, stage 6/7; 16, stage 8/9.

Finally, we investigated CID assembly dynamics in female meiosis in ovaries fixed and stained for CID, using the synaptonemal complex marker C(3)G to identify the oocyte nucleus (Figure 3A) [40]. Quantification of total centromeric CID intensity in oocyte nuclei revealed a 2-fold increase in CID intensity from cystoblasts to stage 8/9 of egg chamber development (Figure 3B). Thus, CID assembly occurs during the pachytene and diplotene stages of prophase I, which last approximately 3 days [41]. Due to reduced antibody penetration at later stages of oocyte development, we were unable to assess whether CID loading continues during later stages of prophase I and beyond.

We conclude that CID assembly in Drosophila male and female meiosis I occurs during prophase and, surprisingly, that loading is gradual and occurs over a period of days. Importantly, this temporal pattern is conserved despite significant differences between male and female meiosis I prophase; although homolog pairing occurs in both, synapsis and recombination only occur in females.

### A Second Phase of CID Assembly in Meiosis in Spermatids

**Figure 4:**
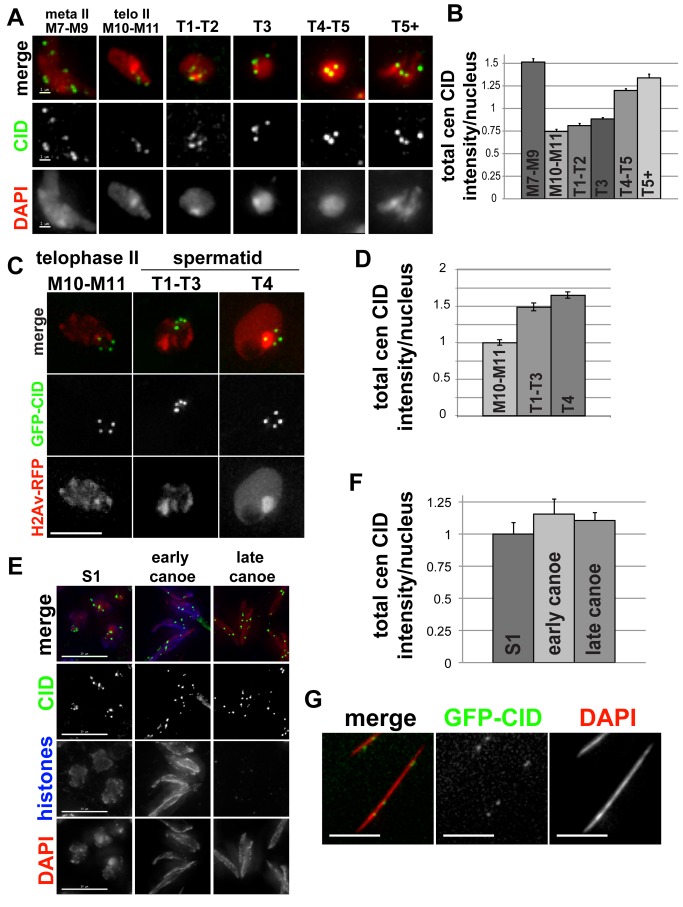
CID assembly in male meiosis II. (A) Changes in the amount of CID at centromeres during meiosis II and in differentiating spermatids. Larval testes were fixed and stained with anti-CID antibody (green), and DNA is stained with DAPI (red). Scale bar: 1 µM. (B) Quantification of total centromeric CID fluorescence intensity per nucleus in meiosis II and in differentiating spermatids. Bars, standard errors. *N* = 799 total cells; 25, M7–M9; 114, M10–M11; 90, T1–T2; 295, T3; 196, T4–T5; 79, maturing spermatids. Note that Figures 2C and 4B are from the same experiment and to the same scale, normalized to the initial S1 intensity value. (C) Live imaging of GFP-CID (green) and H2Av-RFP (red) expression in M10–M11 (telophase II) and differentiating spermatids (T1–T3 and T4). Scale bar: 3 µM. (D) Quantification of total centromeric GFP-CID fluorescence intensity per nucleus from live imaging of stage M10–M11 (telophase II, *n* = 24) and T1–T3 (*n* = 36) and T4 (*n* = 38) spermatids. Bars, standard errors. (E) CID localization on spermatids in stage S1 primary spermatocytes and before (early canoe stage) and after (late canoe stage) protamine exchange in adult testes fixed and stained with anti-CID antibody (green), anti-histone (blue), and DAPI (red). Scale bar: 15 µM. (F) Quantification of total centromeric CID fluorescence intensity per nucleus S1 primary spermatocytes, and early and late canoe stage spermatids in adult testes. Bars, standard errors. Values for early and late canoe are scaled to the S1 value. *N* = 144 total cells; 52, S1; 25, early canoe; 67, late canoe. (G) CID localization in mature spermatozoa. Adult testes from GFP-CID (green) transgenic flies were fixed and stained with DAPI (red). Scale bar: 5 µM.

We next investigated CID assembly dynamics during male meiosis II and subsequent stages of sperm differentiation (Figure 4). In fixed samples, we did not detect any increase in CID intensity between metaphase (stages M7–M9) and telophase (stages M10–M11) of meiosis II; instead, total centromeric CID intensity per nucleus drops by half, as expected due to the segregation of sister chromatids. We observed that total CID intensity increases gradually beginning in T1–T2 spermatid nuclei, after exit from meiosis II, reaching almost a 2-fold increase in spermatids that have initiated differentiation into spermatozoa (T5+ stages) (Figure 4A and 4B; fixed cells from the same experiment presented in Figure 2). Live imaging confirmed that CID levels increase between telophase II and T4 spermatids (Figure 4C and 4D). Although the exact length of stages T1–T5 is not known, it likely occurs over hours to days, because the entire process of spermatid differentiation to mature spermatozoa takes ∼6 days [42]. Thus, similar to observations in prophase I, CID assembly in spermatids is gradual and occurs over an extended time period.

We next investigated if CID is retained on spermatids after gross histone removal in preparation for protamine exchange (Figure 4E and 4F). We observed in adult testes that CID is present at the late canoe stage (after histone removal), consistent with a previous report [35], and that levels are comparable to levels in spermatids at an earlier stage when histones are still present. Furthermore, CID levels after gross histone removal are comparable to levels in S1 stage primary spermatocytes (Figure 4F). To investigate whether CID is retained at even later stages, in mature sperm, which contain highly condensed chromatin that is inaccessible to antibody staining, we fixed and imaged adult testes from transgenic flies expressing GFP-CID. Mature spermatozoa contain four GFP-CID spots that were clearly visible and spaced along the length of the nucleus (Figure 4G). We conclude that CID is retained at centromeres in mature spermatozoa in adults.

**Figure 5:**
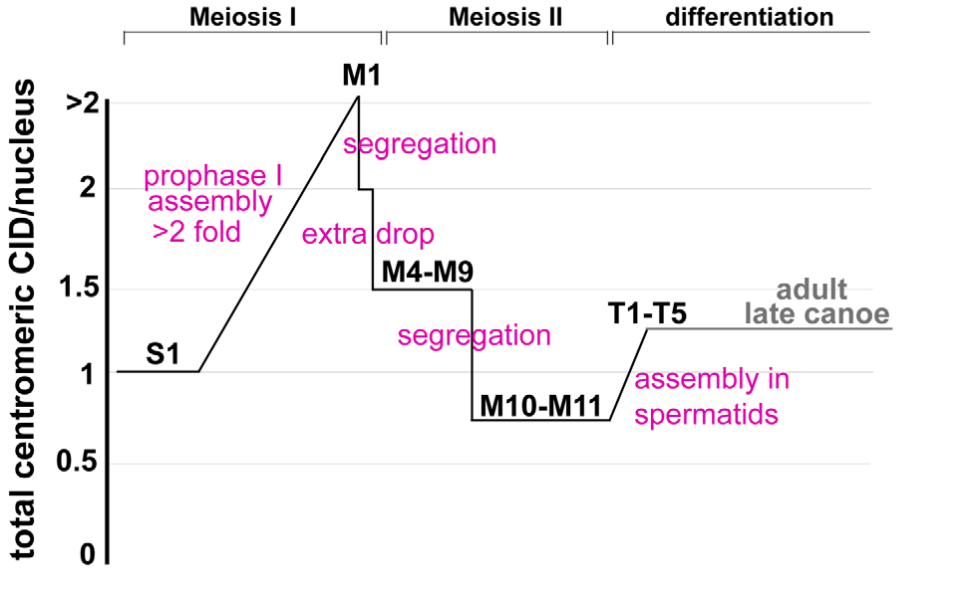
Summary of the timing of CID assembly in male meiosis, showing the net gain or loss in total centromeric CID per nucleus. CID assembly occurs in prophase of meiosis I, resulting in a greater than 2-fold increase in CID at centromeres from S1 to M1 stages. CID drops by half (CID level = 2, relative to starting levels in S1) due to chromosome segregation in meiosis I; additional loss of CID is observed between telophase I and the beginning of meiosis II (CID level = 1.5). CID drops by half due to chromosome segregation in meiosis II with no assembly (CID level = 0.75). A second phase of CID assembly occurs beginning in T1 spermatids and continuing in T2–T5 spermatids in larvae (CID level = 1.25). In larvae, the level of centromeric CID in T5 haploid spermatids is comparable to the level at the S1 stage before the meiotic divisions. In adults, the level of centromeric CID in late canoe stage spermatids is also comparable to the level at the S1 stage.

From our fixed and live analyses, we conclude that after premeiotic S phase there are two phases of CID assembly during male meiosis: first, during prophase of meiosis I, and second, beginning in T1 spermatids after exit from meiosis II (summarized in Figure 5). Our results also demonstrate that CID levels increase by more than 2-fold in prophase I and are surprisingly reduced by greater than half after the first meiotic division and before the onset of meiosis II. Taken together, the amount of CID in haploid spermatids (T5+) is similar to the amount of CID per nucleus at the beginning of meiosis (stage S1) (compare Figures 2C and 4B, showing quantifications from the same experiment, both normalized to the S1 intensity value). Finally, analysis of adult testes reveals that CID levels on haploid mature sperm are also comparable to levels at the S1 stage, before the meiotic divisions.

### CAL1 and CENP-C Levels at Centromeres Decrease as Meiosis Progresses

**Figure 6:**
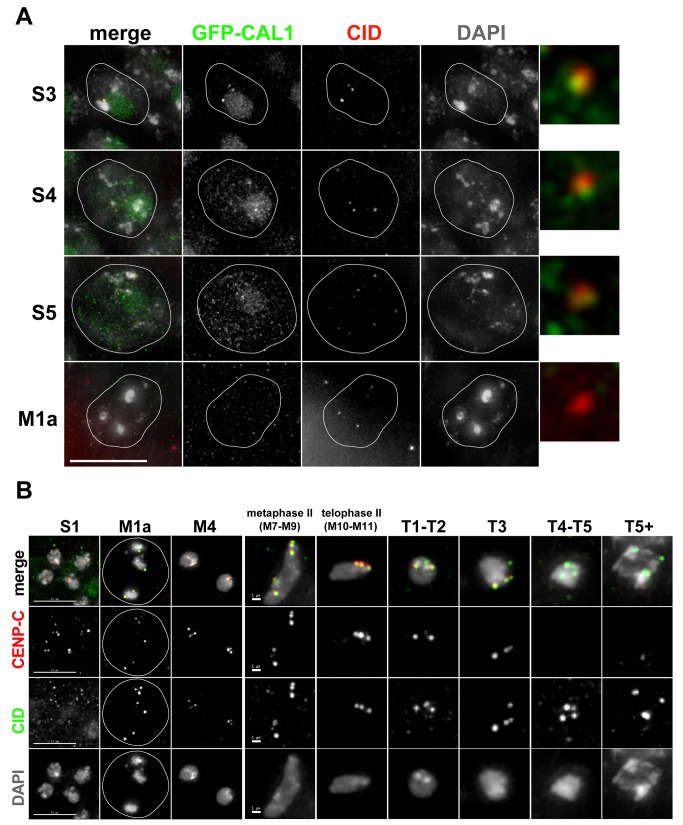
CAL1 and CENP-C localization in meiosis. (A) Fixed imaging of GFP-CAL1 expression/localization in prophase I in larval testes. GFP-CAL1 is localized at centromeres and in the nucleolus in stage S3 and S4 nuclei, but is reduced/delocalized from centromeres by stage S5 and almost undetectable by stage M1a (late prophase I). Colocalization of GFP-CAL1 (green) and CID (red) signals at centromeres are shown in enlarged windows. Outlines of nuclei are circled in white. Scale bar: 10 µM. (B) Fixed cell analysis of CENP-C localization in larval testes. Larval testes were fixed and stained with anti-CENP-C antibody (red), anti-CID antibody (green), and DAPI (gray). CENP-C is present at centromeres at all stages of meiosis from prophase I to telophase II (shown, stages S1, M1a, M4, M7–M9, M10–M11) but is gradually lost from centromeres beginning at T1, coinciding with the time of CID assembly (see Figure 4). CENP-C is absent from centromeres in later stage T4–T5+ spermatids (see Figure S4). Scale bar: 5 µM, S1–M4; 1 µm, M7–M9–T5+.

Both CAL1 and CENP-C are required for CID maintenance and assembly in mitotic cells in flies and cultured cells [24]–[26], but their presence, localization, and function in meiosis are unknown. We stained larval testes from a transgenic fly line expressing GFP-CAL1 [25] with anti-GFP and anti-CID antibodies and observed that GFP-CAL1 localized at centromeres, and also the nucleolus, in cells at the S3 stage of prophase I (Figure 6A) and earlier stages in the germinal proliferation center (Figure S3A). Surprisingly, GFP-CAL1 foci at centromeres are dramatically reduced by the S5 stage and are almost undetectable in nuclei at late prophase I (M1a). Using live imaging, we observe GFP-CAL1 foci in S1–S3-stage nuclei, but not in cells that have completed meiosis I (stage M5) or II (onion stage spermatids). Note that GFP-CAL1 accumulates in the cytoplasm and the nebenkern mitochondrial derivative, respectively, during these stages (Figure S3A). Furthermore, live imaging of female oocytes revealed that GFP-CAL1 foci are present in cystoblast nuclei but are undetectable in stage 4 oocyte nuclei (Figure S3B).

We next determined if CENP-C is localized at centromeres during meiosis by staining larval testes with anti-CENP-C antibody. Similar to GFP-CAL1, CENP-C is visible as discrete foci that colocalize with CID at the S1 stage (Figure 6B). However, distinct from CAL1, CENP-C is present at centromeres through all stages of meiosis I (stages M1a and M4) and II (stages M7–M11) but is gradually lost from centromeres beginning after telophase of meiosis II (M10–M11). CENP-C loss is coincident with the start of post-meiosis II CID assembly (T1–T2 spermatids) and prior to the continued assembly in stages T4 and later (Figure 6B). We also observed that CENP-C is absent from centromeres on individualizing spermatids in adult testes and is localized to structures peripheral to the nucleus in T4–T5 spermatids, then cleared away from nuclei along the elongating axoneme during later stages of maturation (Figure S4).

We conclude that the centromere proteins CAL1, CENP-C, and CID show differential localization patterns during meiosis. CID is present at centromeres throughout meiosis and is retained on mature sperm (Figure 4). In contrast, CAL1 levels at centromeres are dramatically reduced during prophase of meiosis I, coincident with the time of CID loading, and centromeric CAL1 is not visible after late prophase I through the end of spermatogenesis. Finally, CENP-C is not visible at centromeres after meiosis II, during the second phase of CID loading, and in mature sperm.

### CAL1 and CENP-C Are Required for CID Assembly and Chromosome Segregation in Meiosis

**Figure 7:**
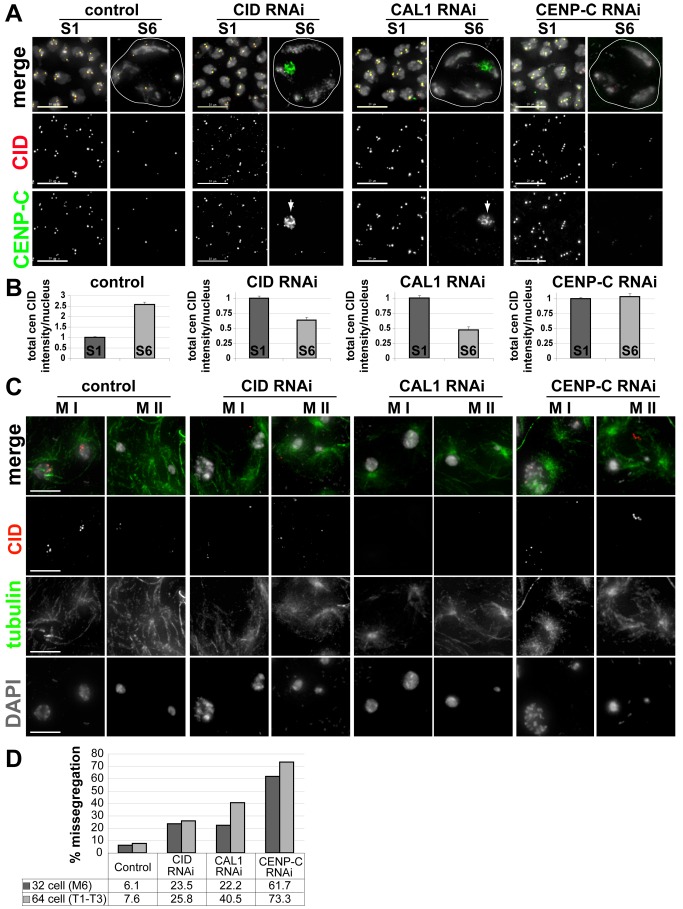
Requirements of CAL1 and CENP-C for CID localization in meiosis. (A) Testes from pre-pupal larvae propagated at 29°C were fixed and stained with anti-CID (red) and anti-CENP-C (green) antibodies, and DAPI (grey). Wild type control, *bam-Gal4-VP-16*; CID-RNAi, *bam-Gal4-VP-16/+*; *UAS-Cid-RNAi*; CAL1 RNAi, *bam-Gal4-VP16/+*; *UAS-Cal1-RNAi*; CENP-C RNAi, *bam-Gal4-VP16/+*; *UAS-Cenp-C-RNAi*. Nuclei in meiotic prophase I, stages S1 and S6, are shown. White arrows indicate CENP-C localization in the nucleolus. Scale bar: 10 µM. (B) Quantitation of total centromeric CID fluorescent intensity per nucleus in wild-type, CID-, CAL1-, and CENP-C-RNAi in stages S1 and S6. For each graph, the S6 is normalized to the S1 value. *N* = control S1; 54, S6; 27, CID-RNAi S1; 92, S6; 52, CAL1-RNAi S1; 112, S6; 5, CENP-C-RNAi S1; 110, S6; 46. (C) Testes from pre-pupal larvae propagated at 29°C were fixed and stained with anti-CID (red) and anti-tubulin (green) antibodies and DAPI (grey). Representative images for chromosome segregation in meiosis I and II in control, CID-, CAL1-, and CENP-C-RNAi are shown. Scale bar: 10 µM. (D) Quantitation of the percentage of chromosome mis-segregation events in control, CID-, CAL1-, or CENP-C RNAi, in 32 cell cysts (M6 stage) after meiosis I and 64 cell cysts (T1–T3 stages) after meiosis II.

Although CAL1, CENP-C, and CID are mutually dependent for centromere localization in both fly cultured cells and embryos [24],[26], the unusual localization patterns observed for CAL1 and CENP-C during meiosis suggested that these proteins may not be essential for CID localization in male meiosis. We depleted CID specifically in larval testes using a *UAS*-*Cid-RNAi* line [43] driven by GAL4 under the control of the *bam* (*bag of marbles*) promoter (*bam-Gal4*), which is repressed in germline stem cells and expressed in spermatogonia at the four-cell stage, after completion of two mitotic divisions [44],[45]. In prepupal testes depleted for CID, CID staining was normal in the S1 primary spermatocytes but was dramatically reduced in nuclei at stage S6 of prophase I compared to *bam-Gal4* controls (Figure 7A and 7B). Additionally, in cells depleted for CID, CENP-C was delocalized from centromeres and accumulated in the nucleolus (Figure 7A, arrow).

To investigate if CAL1 or CENP-C are required for CID localization in meiosis, *UAS*-*Cal1-RNAi* or *UAS-Cenp-C-RNAi* lines [43] were crossed to lines expressing the *bam-Gal4* driver. In prepupal testes depleted for CAL1, centromeric CID levels were normal in S1 primary spermatocytes but were dramatically reduced in nuclei at stage S6 of prophase I, compared to *bam-Gal4* control testes (Figure 7A). Thus, CAL 1 is required for CID assembly in prophase of meiosis I. In prepupal testes with RNAi-depleted CID or CAL1, we also observed an elevated frequency of nuclear mis-segregation after the first (stage M6) and second (stages T1–T3) meiotic divisions (Figure 7C and 7D), indicating that CID and CAL1 are required for normal progression through male meiosis. Additionally, CENP-C was present at centromeres in S1 stage cells depleted for CAL1, but in stage S6 of prophase I was significantly reduced at centromeres and accumulated in the nucleolus, as observed in CID-depleted cells (Figure 7A, arrows). These observations in meiotic cells are consistent with previous reports in cultured mitotic cells, which showed that CAL1 is required for both CID and CENP-C localization and that CENP-C accumulates in the nucleolus in the absence of CAL1 [24]–[26]. We conclude that CAL1 is required for centromeric CID assembly and localization of CENP-C in prophase of meiosis I and proper chromosome segregation in both meiotic phases. It is surprising that CAL1 is required for both meiosis I progression and CID/CENP-C prophase loading and maintenance at centromeres, despite being undetectable at these stages (Figure 6).

RNAi depletion of CENP-C in prepupal testes also resulted in reduced CID localization at centromeres in S6 stage cells (although to a lesser extent than the depletion of either CAL1 or CID), indicating a requirement for CENP-C in CID assembly in prophase (Figure 7A and 7B). IF analysis shows that the reduction in CENP-C levels was comparable after CID-, CAL1-, and CENP-C RNAi depletions; this suggests that CAL1 plays a more major role than CENP-C in CID localization in meiosis. Notably, in T1–T3 spermatids depleted for CAL1 or CENP-C, CID levels at centromeres are low and almost undetectable in the case of CAL1 RNAi (Figure 7C), indicating possible roles for CAL1 and CENP-C in the second phase of meiotic CID assembly. In cells depleted for CENP-C, severe defects in chromosome segregation were still observed after meiosis I and meiosis II (Figure 7C and 7D), even though CID still remained at centromeres at levels higher than observed after CID or CAL1 RNAi depletions (Figure 7C), likely due to the additional role of CENP-C in kinetochore assembly and function. Furthermore, depletion of CENP-C in tissues using the *MTD-Gal4* driver, which is expressed throughout all stages of spermatogenesis and oogenesis [46], shows that it is required for testes and ovary development, presumably due to its essential role in centromere propagation and kinetochore assembly in mitosis (Figure S5).

We conclude that CAL1 and CENP-C are both required for CID assembly in prophase of meiosis I in Drosophila males and for normal progression through spermatogenesis. Thus, despite differences in CID assembly timing between mitosis and meiosis, and the lack of detectable CAL1 during prophase of meiosis I, the assembly protein requirements for meiosis are similar to mitosis. Further investigations are required to determine if CID assembly in meiosis is more dependent on CAL1 than CENP-C, compared to the equal requirements in mitosis [24].

## Discussion

This study reveals a surprising diversity of CID assembly timing in mitotic and meiotic tissues in the fruit fly *Drosophila melanogaster*. During mitosis, CID assembly initiates at late telophase and continues during G1 phase in somatic cells of the larval brain. These results are consistent with the timing and dynamics of CENP-A assembly reported for human cell lines [10],[36],[37] and in general, with centromeric histone deposition outside of S phase, during mitosis and G1 phase. Notably, we observed loading in mitosis occurring at a later mitotic stage (telophase/G1 phase) than previously reported for cultured cells (metaphase) or fly embryos (anaphase) [13],[14]. Interestingly, neuroblast stem cells display a subtle difference between cells derived from the same division; the mother cell, which will continue to act as a stem cell, starts CID loading at centromeres 3–6 min earlier than in the daughter cell that is committed to differentiation. It is currently unclear whether this difference in centromere assembly timing is due to differences in the regulation of mitotic exit between stem and daughter cells or is required for or a response to stem cell propagation mechanisms.

We propose that such differences in timing reflect altered cell cycle regulation in cultured cells compared to animal tissues, and our results emphasize the importance of validating cell culture findings in animal models. It is important to note that despite similarities to the timing observed in human cultured cells (late telophase/G1 phase) [10], our results in Drosophila raise questions about whether the analysis of cultured cells in humans and other species reflects the timing of CENP-A assembly in the organism.

**Figure 8:**
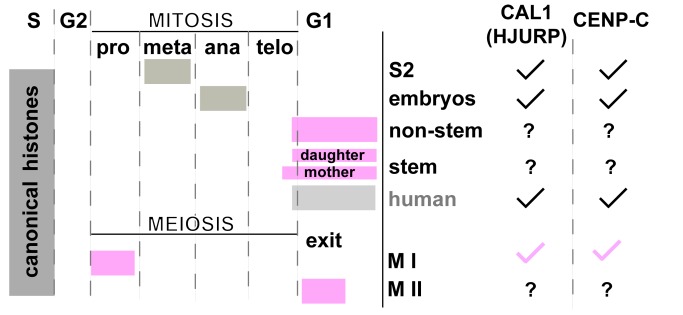
Timing and requirements for CID assembly in mitosis and meiosis. While newly synthesized canonical histones are assembled during DNA replication in S phase, in most organisms the centromeric histone CENP-A is assembled outside of S phase, in mitosis or G1 phase. Drosophila CID is assembled at metaphase in cultured S2 cells [13], at anaphase in the syncytial divisions in embryos [14], and at telophase/G1 phase in larval brain nonstem and neuroblast stem cells (this study). In neuroblasts, CID loading in the mother stem cell precedes loading in the daughter cell (this study). In human mitotic HeLa cells in culture, CENP-A is assembled at late telophase/early G1 phase [10]. In meiosis in flies, CID is assembled at centromeres during prophase of meiosis I and after exit from meiosis II in spermatids (this study). The putative HJURP functional homolog in flies, CAL1, is required for CID loading in S2 cultured cells, in embryos, and in prophase of meiosis I, while HJURP is required for CENP-A loading in human cells [16],[17]. CENP-C is required for CID assembly in S2 cultured cells, embryos [24],[26], and in meiosis (this study). The timing of CENP-A assembly in somatic (stem or nonstem) cells in tissues, in embryonic or meiotic divisions in mammals is currently unknown.

Our results also show that the cell cycle timing for CID assembly in meiosis differs from mitosis (Figure 8). In male meiosis, there are two phases of CID assembly, at prophase of meiosis I and after exit from meiosis II, and two phases of chromosome segregation, resulting in haploid spermatids with nuclear CID levels equivalent to those observed at the beginning of meiosis (see Figure 5). In meiosis in Drosophila females, CID assembly also occurs during prophase of meiosis I. Assembly in prophase provides another example of the restriction of CID assembly to a specific part of the cell cycle outside of S phase, but has not been observed previously in mitotic tissues or cultured cells from other organisms. It is also important to note that meiotic prophase in both male and female Drosophila occurs over days, indicating that CID assembly is gradual over this extended time period. Such slow assembly dynamics are unexpected, given that until now studies in mitotic cells indicate that CENP-A assembly is completed in the order of minutes to hours [10],[13],[14],[36],[37]. How CID assembly is first initiated and then continues over such extended time periods awaits further investigation.

It is likely that cell cycle regulators control CID assembly in meiosis as they do in mitosis. For example, a recent study showed that CDK activity inhibits CENP-A assembly in human cells and that blocking CDK activity results in precocious loading in S and G2 phases [27]. Cyclin A is degraded during late prophase of meiosis I [47]. This is consistent with the observed burst in CID assembly during a 10-min time window of late prophase/early prometaphase I, and our previous demonstration that Cyclin A degradation is required for mitotic CID assembly [13]. However, CID assembly also occurs before Cyclin A degradation in meiosis I, implying that other unknown mechanisms initiate and continue assembly prior to late prophase I. Additionally, CID is not loaded between meiosis I and II, even though Cyclin A levels remain low. Instead, the partial degradation of Cyclin B to an intermediate level after meiosis I, which allows for spindle destruction but prevents a second round of DNA synthesis [48], could inhibit CID assembly between meiosis I and II. Moreover, the slow degradation of Cyclin B at the end of meiosis II [49] could contribute to the gradual CID loading in spermatids, as the second phase of CID assembly after meiotic exit is more similar in terms of cell cycle regulation to the telophase/G1 loading observed in mitotic tissues in the animal (this study) and in human cells in culture [10]. However, we also observed that CID assembly occurs in prophase of meiosis I, when Cyclin B levels are high, but does not occur between meiosis I and II, despite low Cyclin A levels. This suggests that CID assembly in meiosis is regulated by other mechanisms in addition to the inhibition of Cyclin/CDK activities, as proposed for mammalian cells [27].

Another striking observation from this study is that during meiosis I, CID assembly occurs prior to chromosome segregation, whereas most mitotic cells previously studied proceed through most of mitosis with half the maximal amount of CID at centromeres [10],[13],[14],[27]. In addition, we observed a greater than 2-fold increase in CID intensity at centromeres during prophase, even though a 2-fold increase would be sufficient to compensate for CID dilution in premeiotic S phase. What is the role, if any, of an increased level of CID at centromeres during the first meiotic division? In meiosis I, bivalent sister chromatid kinetochores are mono-oriented, instead of bi-oriented as they are in mitosis and meiosis II; combined with the maintenance of sister cohesion at centromeres, this ensures that homologs, and not sisters, segregate during meiosis I [48],[50]. We speculate that extra CID may be required during the first meiotic division to assemble or maintain mono-oriented kinetochores and microtubule attachments. This hypothesis could also be extended to incorporate the surprising decrease in CID levels observed between the end of meiosis I and the beginning of meiosis II. Loss of CENP-A during normal cell divisions has only previously been observed as accompanying DNA replication and nucleosome segregation in S phase, events that do not occur between meiosis I and II. Thus, it is tempting to speculate that the additional loss of CID after meiosis I could contribute to the currently unknown mechanism responsible for reorganization of kinetochores from mono- to bi-orientation in preparation for meiosis II.

Using targeted RNAi depletion of centromeric proteins during Drosophila male meiosis, we find that both CAL1 and CENP-C are required for CID assembly in prophase of meiosis I. This is consistent with previous observations in mitotic cells, where CAL1, CENP-C, and CID are mutually dependent on each other for centromere localization [24],[26]. We also find that depletion of CAL1 or CID in larval testes results in CENP-C delocalization from centromeres and sequestration in the nucleolus, again similar to observations in mitosis [24], possibly because it is no longer in a stable complex with CID or CAL1. Our results also show that reduced CAL1 or CENP-C expression results in defective chromosome segregation and that both are required for normal progression through male meiosis. Our finding that T1–T3 spermatids depleted for CAL1 or CENP-C have reduced CID at centromeres (although to a lesser extent in the case of CENP-C depletion) also suggests that CAL1 and CENP-C are required for CID assembly during the second phase of loading in spermatids. However, given that cells with reduced CAL1 or CID already show major chromosome segregation defects after meiosis I, meiosis-specific GAL4 drivers active in later stages of meiosis and spermatogenesis, which are currently lacking [51], are required to directly assay the requirements for CAL1 and CENP-C in the second phase of CID assembly or during fertilization. Requirements for CAL1 and CENP-C in both phases of meiotic CID assembly are surprising, given that centromeric CAL1 levels are greatly reduced during prophase I and at later stages of spermatogenesis and that CENP-C is not localized to centromeres after meiosis II. One intriguing possibility is that CID assembly requires CAL1 and CENP-C removal from centromeres.

Another key observation from our study is the retention of CID at centromeres on mature spermatozoa in spite of an extensive period of chromatin remodeling and histone–protamine exchange during spermatocyte maturation [52],[53]. How CID is protected from histone removal prior to protamine exchange at centromeres remains to be investigated. It is possible that the local chromatin environment at centromeres is refractory to protamine exchange or that additional proteins present at centromeres could provide protection. Because fusion of male and female pronuclei does not occur until telophase of the first zygotic division [54], it is likely that paternal CID at centromeres is required for kinetochore formation and spindle attachment to paternal chromosomes. The amount of paternal CID at centromeres could be critical for the successful epigenetic inheritance of centromere identity and for the viability of the embryo, if paternal CID is diluted during subsequent zygotic divisions. Alternatively, maternal CID could compensate for a reduced level of CID on sperm or establish de novo centromeres on paternal chromosomes. Whatever the mechanism of CID maintenance in the zygote, the regulation of CID assembly on sperm is likely to prove very important in the transmission of epigenetic information and centromere specification into the next generation.

## Materials and Methods

### Drosophila Stocks

Flies were grown at 25°C on standard medium. Transgenic fly lines expressing GFP-CID and H2Av-RFP were a gift from S. Heidmann [14], and GFP-CAL1 lines were provided by C. Lehner [25]. RNAi lines used were: *UAS-CID-RNAi* (VDRC #102090), *UAS-CAL1-RNAi* (VDRC # 45248), and *UAS-CENP-C-RNAi* (TRiP #34692). The *bam-Gal4* (*w;; bam-Gal4-VP16, UAS-dcr2*) stock was kindly provided by M. Fuller. *MTD-Gal4* stock (# 31777) was purchased from the Bloomington Stock Center. The efficiency of RNAi depletion was enhanced by expression of *dicer2* (M. Fuller) and propagation at 29°C [43],[55]. *y^+^ry^+^* flies were used as wild-type for fixed analyses.

### Immunostaining and Fluorescence Microscopy

Dissection, fixation, and immunostaining of larval and adult testes [38] and oocytes [56] were performed as described previously. Primary antibodies diluted in PBST/FBS were incubated overnight at 4°C. Larval and adult samples were stained with a rabbit anti-CID antibody (Lake Placid, 1∶500), guinea pig anti-CENP-C polyclonal antibody (1∶500) [24], mouse anti-tubulin (Sigma T6199, 1∶100), mouse anti-pan-histone (including histone H1) (Chemicon,1∶150), and mouse anti-GFP (Abcam ab1218, 1∶100). The slides were washed twice for 5 min in PBST and once for 5 min in 1× PBS. All samples were incubated with secondary antibodies (Alexa conjugates from Molecular Probes: goat anti-mouse 546, goat anti-rabbit 488, and goat anti-guinea pig 647) for 1 h at room temperature at a 1∶500 dilution, washed twice for 5 min in PBST, rinsed in 1× PBS, incubated 5 min with 1 µM DAPI in 1× PBS, and washed 5 min in 1× PBS. Prolong Gold antifade reagent (Molecular Probes) was added, and slides were sealed with a coverslip. GFP-CID adult testes were fixed, incubated with DAPI, washed, mounted as described above, and immediately imaged. Ovaries were stained with mouse anti-C(3)G antibody (1∶500) [57] and rabbit anti-CID (1∶200). After overnight incubation at 4°C with primary antibodies, tissues were washed three times for 15 min in PBST. Samples were incubated with secondary antibodies (described above) for 4 h at room temperature, then washed three times for 30 min in PBST, incubated 5 min with 1 µM DAPI in 1× PBS, washed for 5 min in 1× PBS, and mounted on slides as described above. Larval brains were incubated with 5 µM EdU (Invitrogen) for 15 min at room temperature. All images were taken using a DeltaVision Elite microscope system (Applied Precision). A total of 20–30 *z* sections at 0.2 µM were taken for each image at a constant exposure time. Raw images were deconvolved using SoftWorx (Applied Precision) using conserved ratio, five cycles, and medium noise filtering. Quick projections of images were created in SoftWorx using maximum intensity. Images were uniformly scaled in Photoshop.

### Live Imaging

Live imaging of larval testes and ovaries was performed based on the methods described in [58]. Larval testes were dissected in Schneider's medium (Invitrogen) supplemented with 200 µg/ml bovine insulin and were placed in a small drop of the same medium on a glass-bottomed dish (P35G-1.0-14-C, MatTeck) containing a wet Kim-wipe for humidification. Testes were disrupted using a fine tungsten needle and the dish was covered before imaging. Live imaging of larval brains was carried out according to [59]. Imaging was carried out using a DeltaVision Elite microscope system. A total of 20–30 *z* sections at 0.2 µM were collected per time point at a constant exposure time. Images were deconvolved using SoftWorks.

### Quantification Methods

Deconvolved image files from a single slide that were not scaled or projected (.dv format) were analyzed using a script measuring the total fluorescence intensity of CID foci within a single nucleus. Image analysis software was designed with Matlab (MathWorks Inc, Natick, MA) and DIPimage (image processing toolbox for Matlab, Delft University of Technology, the Netherlands). Nuclei were segmented in 3-D using local thresholding of DAPI followed by a watershed algorithm to separate touching nuclei, resulting in a very accurate 3-D volume for each nucleus. Background CID signal was obtained by computing the average pixel intensity of that signal inside nuclei. A wavelet morphological filter was used to enhance intensity peaks of individual centromere foci in the nuclei while reducing noise from nonspecific signals [60]. The volumes of centromeres were then identified by applying a constant threshold on the wavelet filtered image (k-value = 5). The average total intensity of background subtracted CID signal per nucleus was then computed for each class. Thus, we define the total CID fluorescent intensity per nucleus as the total background-corrected 3-D pixel intensity of all foci in a single nucleus. For fixed samples, the mitotic or meiotic stage of each nucleus was classified manually. Average values for each class were scaled by dividing by the average interphase value for larval brain nonstem mitotic cells, the stage S1 value for male meiotic stages, and the cystoblast value for female meiotic stages. Therefore, a value above 1 reflects an increase in CID fluorescent intensity, and a value below 1 reflects a decrease in CID fluorescent intensity with respect to normalized values for each cell stage.

Live movies were analyzed using image-processing modules from the open-source application Fiji [61] controlled with a custom Java code. Total signal intensity for foci pixels inside a selected cell was computed for every frame and normalized to total foci signal intensity at the first frame. The mean background intensity was computed from a background region of interest (ROI) selected by the user. We set the threshold intensity at three times the mean background intensity. Any values above this threshold value inside the selected cell ROI (also manually set by the user) were classified as foci pixels. To correct for fluorophore bleaching at later time points, we computed the average signal intensity for all the foci in each image. We assumed that the observed decrease in mean signal intensity reflects the decrease in fluorophore signal due to bleaching. The values obtained were thus normalized to the average foci intensity at the first time point. The bleaching-corrected total intensity values for the foci pixels were computed as: 
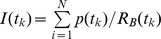
, where *I* (*tk*) is the total intensity value at time *tk*, *p*(*tk*) is the intensity value of a foci pixel inside the cell ROI, and *RB* (*tk*) is the normalized bleaching ratio.

## Supporting Information

Figure S1 Mitotic CID assembly in telophase/G1 phase. (A) Changes in the amount of CID at centromeres at anaphase and early S phase in nonstem brain cells. Larval brains were incubated with EdU to label replicating cells (green) and were fixed and stained with anti-CID antibody (red), and DNA is stained with DAPI (blue). Scale bar: 5 µM. Graph shows total centromeric CID fluorescent intensity per nucleus at anaphase (*n* = 12) and early S phase (*n* = 12). A greater than 2-fold increase is observed in early S phase, due to reduced antibody penetration at anaphase. Bars represent standard errors. (B) Live imaging of GFP-CID (green) and H2Av-RFP (red) in a dividing neuroblast stem cell in the larval brain. Daughter (D, upper) and mother (M, lower) cells are circled, and centromeres in the daughter nucleus at anaphase B and telophase/G1 phase are shown in enlarged windows. Time elapsed from metaphase to early G1 phase is shown in minutes. Scale bar: 10 µM. (C) A neuroblast stem cell in the larval brain fixed and stained with anti-CID (green) and DAPI (red). M = mother cell and D = daughter cells are circled. Scale bar: 10 µM.
(TIF)

Figure S2 No CID assembly during anaphase and telophase of meiosis I. (A) Live imaging of GFP-CID (green) and H2Av-RFP (red) expression in larval testes showing a cell diving by meiosis I. Time elapsed is shown in minutes. Scale bar: 15 µM. (B) Quantification of total centromeric GFP-CID intensity per nucleus during metaphase I and anaphase I A (*n* = 42, chromatin still visible as a single mass), anaphase I B (*n* = 8, two separate chromatin masses visible), and telophase (*n* = 33). Bars represent standard errors.
(TIF)

Figure S3 Live imaging of GFP-CAL1 localization in testes and ovaries. (A) Live imaging of GFP-CAL1 (green) expression/localization in larval testes also expressing H2Av-RFP (red) in the germinal proliferation center, stages S3 and S6 of prophase I, stage M5 (interphase II), and in onion stage spermatids. Scale bar: 15 µM. (B) Live imaging of GFP-CAL1 (green) expression/localization in ovaries also expressing H2Av-RFP (red) in a cystoblast and stage 4 oocyte. Scale bar: 2 µM.
(TIF)

Figure S4 CENP-C removal from mature spermatozoa. Adult testes were fixed and stained with anti-CENP-C antibody (green) and DAPI (red). T4–T5 spermatids (after meiosis II) and individualizing spermatids are shown. Scale bar: 15 µM.
(TIF)

Figure S5 Ovary and testis development is arrested in flies expressing CENP-C RNAi under the control of *MTD-Gal4* driver. (A) Ovaries of wild-type females grow to normal size and produce mature eggs, while ovaries expressing *UAS-Cenp-C-RNAi* and *MTD-Gal4* arrest development at an early stage. (B) In males expressing *UAS-Cenp-C-RNAi* and *MTD-Gal4*, mature testes do not develop (accessory glands are visible). For each GAL4/RNAi cross, flies were raised at 25°C. Female flies were dissected either the same day as eclosion or were yeast-fed at 25°C for 4 d before dissection. Male flies were dissected 2 d after eclosion. Tissues were dissected in PBS and images were taken at 4× magnification using a camera attached to a dissecting scope.
(TIF)

Movie S1 Movie of CID assembly at telophase/early G1 phase of mitosis in a larval brain nonstem cell (somatic). GFP-CID is shown in green and H2Av-RFP in red. Each frame is a 6-min time point.
(MOV)

Movie S2 Movie of CID assembly at telophase/early G1 phase of mitosis in a larval brain neuroblast stem cell (somatic). GFP-CID is shown in green and H2Av-RFP in red. Each frame is a 3-min time point.
(MOV)

Movie S3 Movie of CID assembly at early prometaphase of meiosis I in larval testes. GFP-CID is shown in green and H2Av-RFP in red. Each frame is a 10-min time point.
(MOV)

Movie S4 Movie of a cell dividing in meiosis I from metaphase I to telophase I in larval testes. GFP-CID is shown in green and H2Av-RFP in red. Each frame is a 5-min time point.
(MOV)
