# Supplementary figures and images for: The Cell Cycle Timing of Centromeric Chromatin Assembly in Drosophila Meiosis Is Distinct from Mitosis Yet Requires CAL1 and CENP-C
Source: PLoS Biol. 2012 Dec 27;10(12):e1001460. doi: 10.1371/journal.pbio.1001460 (PMC3531500; doi:10.1371/journal.pbio.1001460)

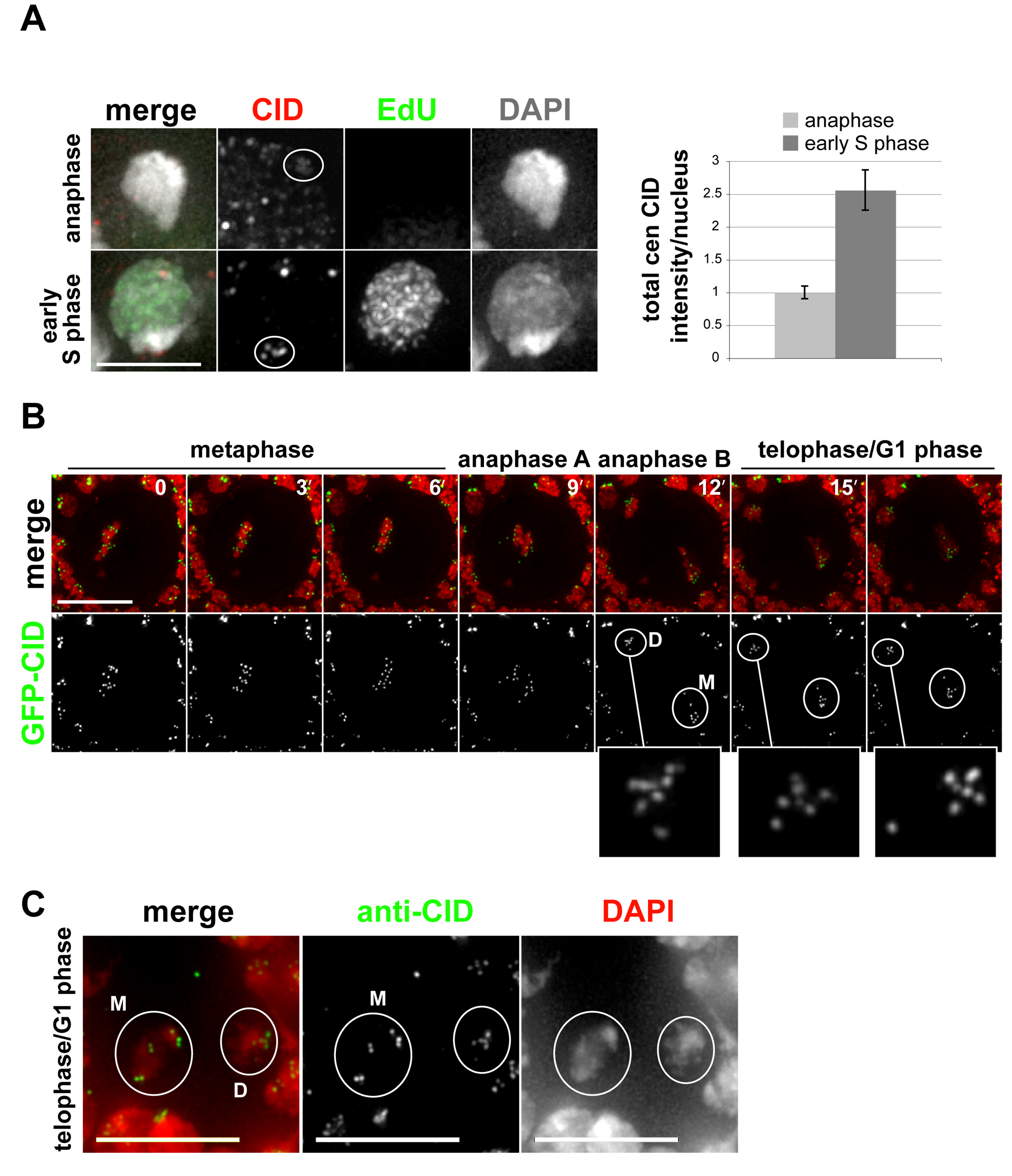

Supplement: Figure S1 — Mitotic CID assembly in telophase/G1 phase. (A) Changes in the amount of CID at centromeres at anaphase and early S phase in nonstem brain cells. Larval brains were incubated with EdU to label replicating cells (green) and were fixed and stained with anti-CID antibody (red), and DNA is stained with DAPI (blue). Scale bar: 5 µM. Graph shows total centromeric CID fluorescent intensity per nucleus at anaphase (n = 12) and early S phase (n = 12). A greater than 2-fold increase is observed in early S phase, due to reduced antibody penetration at anaphase. Bars represent standard errors. (B) Live imaging of GFP-CID (green) and H2Av-RFP (red) in a dividing neuroblast stem cell in the larval brain. Daughter (D, upper) and mother (M, lower) cells are circled, and centromeres in the daughter nucleus at anaphase B and telophase/G1 phase are shown in enlarged windows. Time elapsed from metaphase to early G1 phase is shown in minutes. Scale bar: 10 µM. (C) A neuroblast stem cell in the larval brain fixed and stained with anti-CID (green) and DAPI (red). M = mother cell and D = daughter cells are circled. Scale bar: 10 µM. (TIF) [file pbio.1001460.s001.tif]

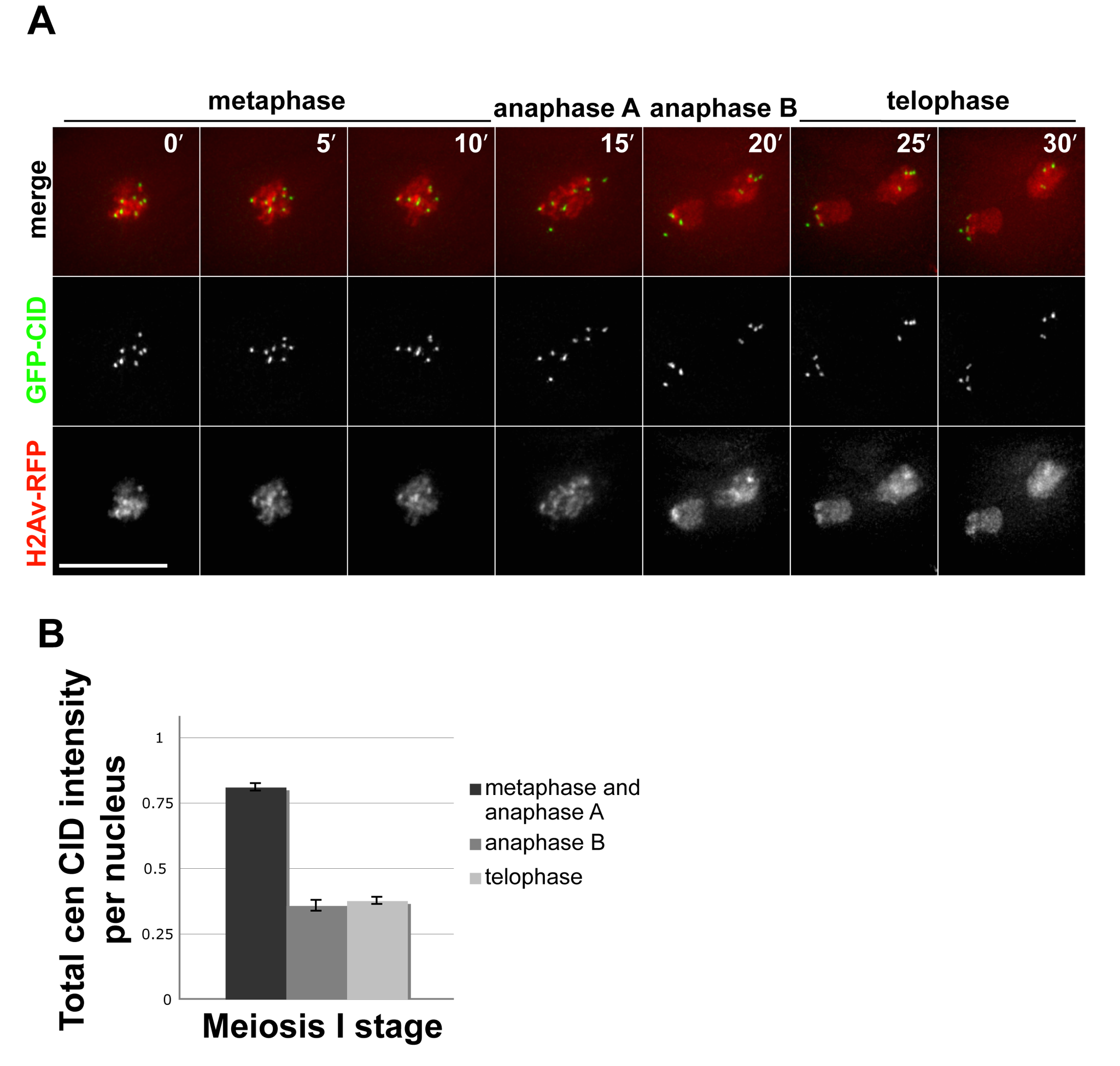

Supplement: Figure S2 — No CID assembly during anaphase and telophase of meiosis I. (A) Live imaging of GFP-CID (green) and H2Av-RFP (red) expression in larval testes showing a cell diving by meiosis I. Time elapsed is shown in minutes. Scale bar: 15 µM. (B) Quantification of total centromeric GFP-CID intensity per nucleus during metaphase I and anaphase I A (n = 42, chromatin still visible as a single mass), anaphase I B (n = 8, two separate chromatin masses visible), and telophase (n = 33). Bars represent standard errors. (TIF) [file pbio.1001460.s002.tif]

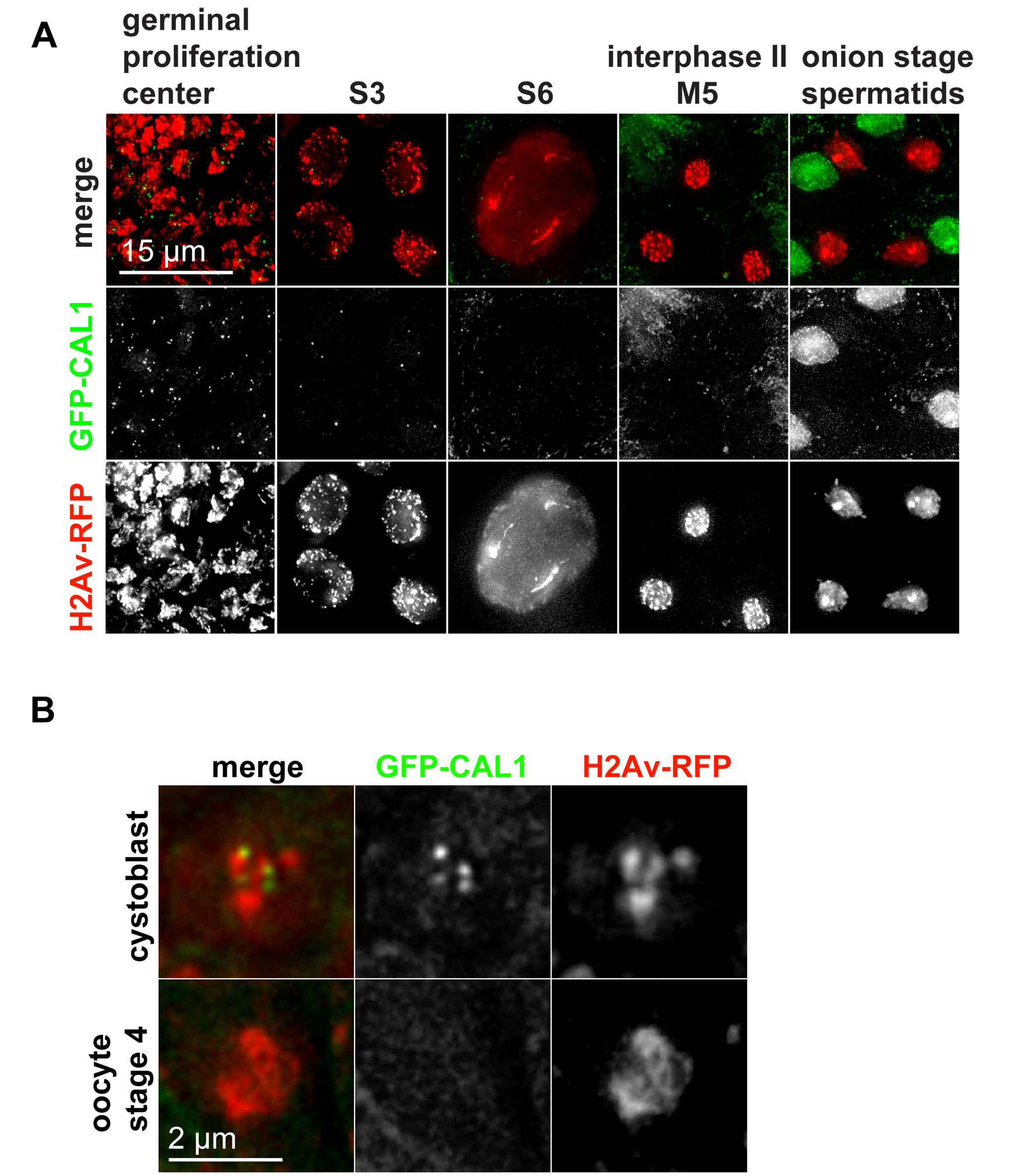

Supplement: Figure S3 — Live imaging of GFP-CAL1 localization in testes and ovaries. (A) Live imaging of GFP-CAL1 (green) expression/localization in larval testes also expressing H2Av-RFP (red) in the germinal proliferation center, stages S3 and S6 of prophase I, stage M5 (interphase II), and in onion stage spermatids. Scale bar: 15 µM. (B) Live imaging of GFP-CAL1 (green) expression/localization in ovaries also expressing H2Av-RFP (red) in a cystoblast and stage 4 oocyte. Scale bar: 2 µM. (TIF) [file pbio.1001460.s003.tif]

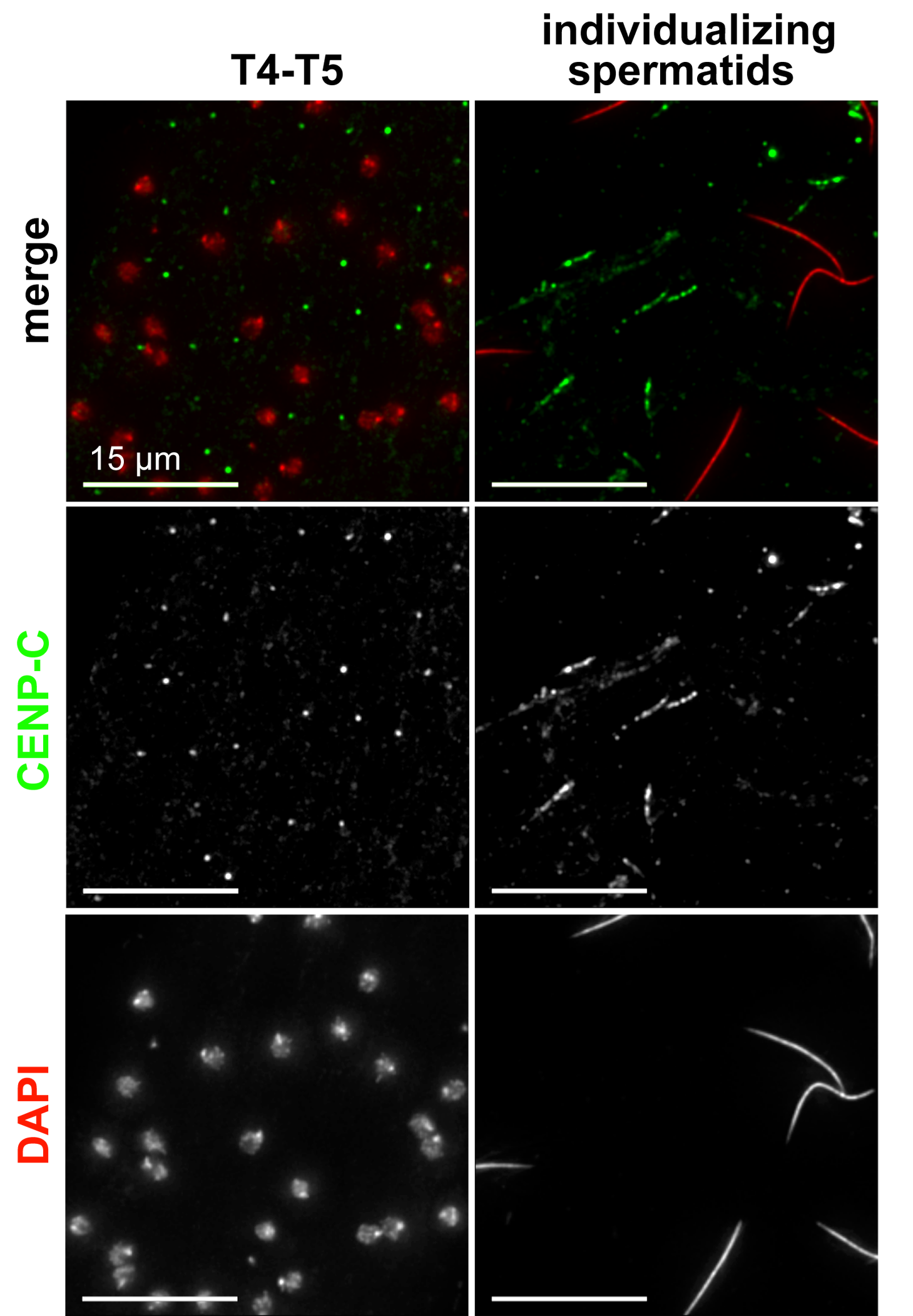

Supplement: Figure S4 — CENP-C removal from mature spermatozoa. Adult testes were fixed and stained with anti-CENP-C antibody (green) and DAPI (red). T4–T5 spermatids (after meiosis II) and individualizing spermatids are shown. Scale bar: 15 µM. (TIF) [file pbio.1001460.s004.tif]

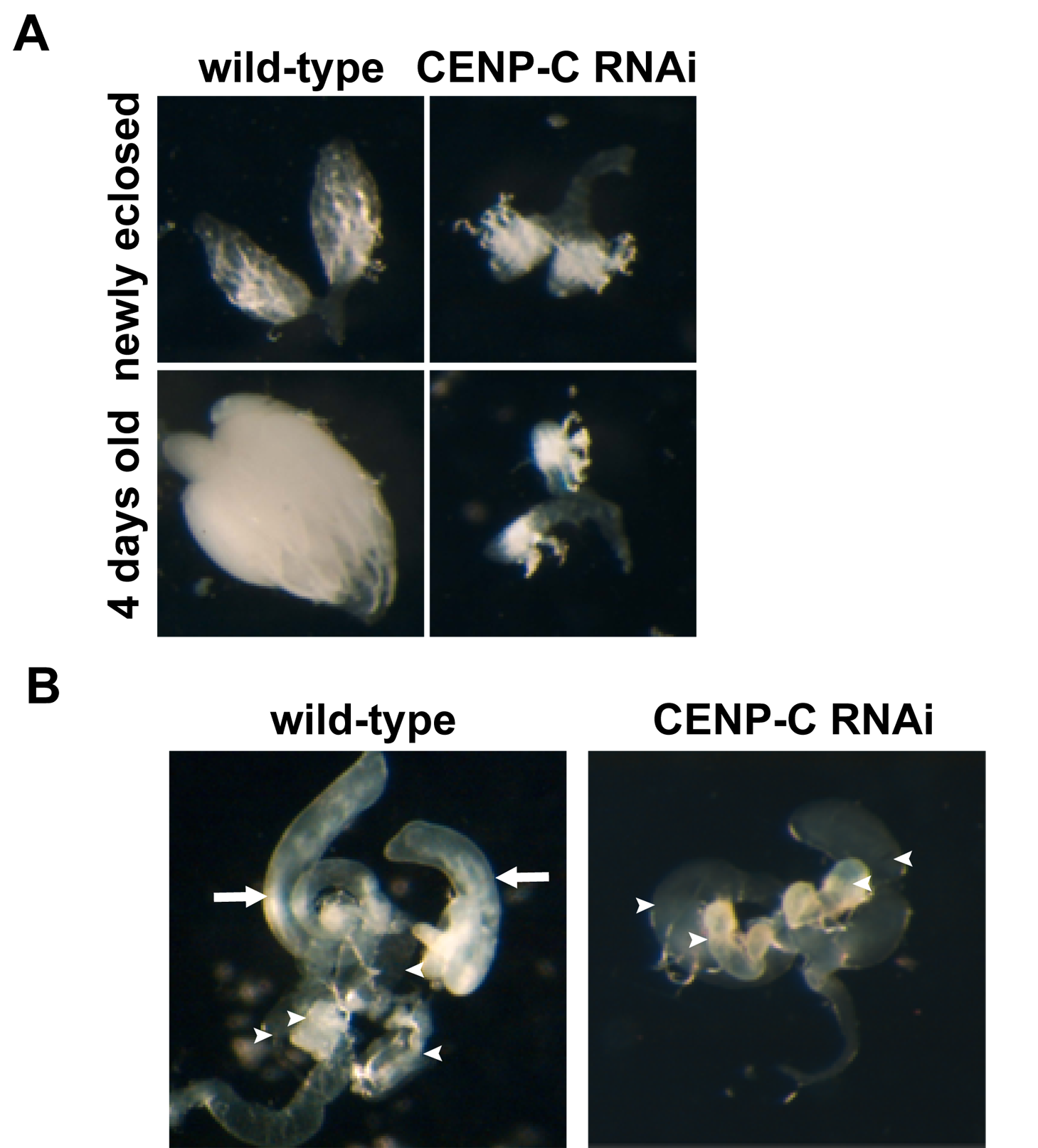

Supplement: Figure S5 — Ovary and testis development is arrested in flies expressing CENP-C RNAi under the control of MTD-Gal4 driver. (A) Ovaries of wild-type females grow to normal size and produce mature eggs, while ovaries expressing UAS-Cenp-C-RNAi and MTD-Gal4 arrest development at an early stage. (B) In males expressing UAS-Cenp-C-RNAi and MTD-Gal4, mature testes do not develop (accessory glands are visible). For each GAL4/RNAi cross, flies were raised at 25°C. Female flies were dissected either the same day as eclosion or were yeast-fed at 25°C for 4 d before dissection. Male flies were dissected 2 d after eclosion. Tissues were dissected in PBS and images were taken at 4× magnification using a camera attached to a dissecting scope. (TIF) [file pbio.1001460.s005.tif]
